# Impact of an Antiresonant Oxide Island on the Lasing of Lateral Modes in VCSELs

**DOI:** 10.3390/ma13092195

**Published:** 2020-05-11

**Authors:** Marta Więckowska, Robert P. Sarzała, Rafał Ledzion, Maciej Dems

**Affiliations:** Institute of Physics, Lodz University of Technology, ul. Wólczańska 219, 90-924 Łódź, Poland; marta.wieckowska@dokt.p.lodz.pl (M.W.); robert.sarzala@p.lodz.pl (R.P.S.); rafal.ledzion@p.lodz.pl (R.L.)

**Keywords:** VCSEL, ARROW, oxidation, antiresonance, waveguiding, optical modeling, electrical modeling

## Abstract

Use of antiresonant structures is a proven, efficient method of improving lateral mode selectivity in VCSELs. In this paper, we analyze the impact of a low-refractive antiresonant oxide island buried in a top VCSEL mirror on the lasing conditions of lateral modes of different orders. By performing comprehensive thermal, electrical, and optical numerical analysis of the VCSEL device, we show the impact of the size and location of the oxide island on the current-crowding effect and compute threshold currents for various lateral modes. If the island is placed close to the cavity, the threshold shows strong oscillations, which for moderate island distances can be tuned to increase the side mode discrimination. We are therefore able to pinpoint the most important factors influencing mode discrimination and to identify oxide island parameters capable of providing single-lateral-mode emission.

## 1. Introduction

Designing high-power vertical-cavity surface-emitting lasers (VCSELs) that operate on a single lateral optical mode is still a challenge. A common way to achieve single-mode emission is to make a relatively small electrical aperture (around 4 µm) [1,2,3]. However, small apertures result in increased differential resistivity, higher current densities, and stronger heating [4]. These effects limit the laser output power to less than 1 mW [5,6,7]. Various approaches have been considered to overcome this limitation, like use of graphene-bubble dielectric distributed Bragg reflectors (DBRs) [8], double cavities [4,9], shallow relief [10] zinc-diffusion with oxide-relief [11,12], and grating couplers [13,14,15]. One of the promising possibilities is the application of antiresonant reflecting optical waveguides (ARROWs) within the VCSEL structure [16,17,18,19]. Several such approaches have been successful, including a simplified version of the ARROW structure (S-ARROW) containing a low-index core surrounded by a single high-index ring. With this structure, substantial mode discrimination has been observed for 980 nm VCSELs with a core diameter of 8–12 µm [20,21,22,23,24,25,26].

In a previous work [27], we introduced an S-ARROW structure directly into the VCSEL cavity, inside the device aperture (i.e., on its axis). The structure was in the form of an oxide island manufactured with a planar oxidation technology [28,29]. We showed that such an oxide island can have a strong impact on the lateral modes in the VCSEL. Their optical losses do not change monotonically with the island size, but are of an oscillatory nature. We proved that these oscillations are caused by the distorting effect of the island, which has a low-refractive-index, on the spatial profiles of the modes. For low island sizes, the modes have a tendency to focus on the high-effective-index region outside its radius, while with larger islands they are confined within it. To our knowledge, this was the first demonstration of the impact of an S-ARROW structure buried in the laser. Because the oscillations were different for modes of different orders, we identified a set of parameters for which the optical losses of one of the modes were significantly smaller than those of the others. For those parameters, the investigated laser had strong potential for being a single-mode device.

The main drawback of the analysis presented in [27] was the fact that it was a purely cold-cavity analysis, which did not consider the impact of the oxide island on the current flow. In the present article, we address this gap by investigating not only the optical modes but also the current flow and the resulting temperature distribution inside the laser structure. As a consequence, we can propose a design for a 850 nm VCSEL with improved modal discrimination, capable of single-lateral-mode high-power emission.

## 2. Methodology

In order to investigate the impact of the oxide island on the thermo-electric properties the analyzed device, we performed a rigorous finite-element analysis of the current flow coupled with heat dissipation. Details of the numerical model used for this purpose are presented elsewhere [30], so we provide only a summary here. In general, the computations are based on the finite-element method with both electric and thermal conductivities dependent on temperature. This makes it necessary to perform the calculations in a self-consistent loop, in order to determine stable current and temperature distributions.

Since, due to the presence of a semiconductor junction, there is no simple linear relation between the voltage and the current [31,32], the determination of the current density distribution also requires an iterative procedure. The number of iterations is usually significantly larger than the number of repetitions due to the thermal effects. Therefore, the thermal computations can be intermixed with the electrical computations and it is sufficient that the temperature distribution is updated once every 10–20 electrical iterations [33].

Once the temperature and current density distribution has been computed and the electric carrier concentration has been determined, we can compute optical gain distribution in the active region. This translates into the complex refractive index in the cavity and allows the hot-cavity optical modes to be determined. We also treat the refractive index as a complex quantity everywhere outside the active region, with the imaginary part resulting from material absorption. The already known temperature distribution allows consideration of the thermal lensing effect. However, we show later that this effect is significant only at high currents, while for low and moderate currents, its influence on the optical modes is minor compared to that of the oxide island.

The optical calculations are performed using both a fully vector plane-wave admittance method [34] and an effective-frequency method [35]. Although the latter is a scalar method and neglects the vector properties of light (it is therefore only capable of analyzing LP modes), we have verified that both methods yield consistent results. We determine the thresholds of all modes by requiring their net optical loss to be equal to zero (i.e., we do not need to compute the overlap integrals to approximate the threshold gain). Above the threshold, the carriers distribution in the active region is adjusted by considering the spatial hole burning effect and the optical modes are recomputed in a self-consistent loop in order to determine the emitted power.

## 3. Analyzed Structure

**Figure 1:**
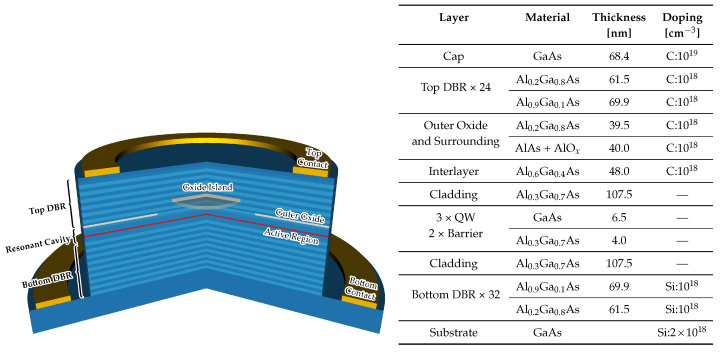
Schematic diagram and layers summary of the analyzed VCSEL. In the image, the active region is marked in red and there are two oxide layers (marked in beige). The outer layer, manufactured classically, is at the edge of the cavity, and the oxide island (shown in full) is in one of the ${\mathrm{Al}}_{0.9}$${\mathrm{Ga}}_{0.1}$As layers in the top DBR.

The investigated device, shown schematically in Figure 1 is an 850 nm VCSEL with the active region consisting of three 6.5-nm-thick active GaAs quantum wells separated by 4-nm-thick AlGaAs barriers. Inside the resonant cavity, with a thickness equal to 1.5 wavelengths of emitted radiation, there is a 40-nm-thick AlAs layer oxidized externally to create a 10 µm electrical aperture for current confinement. Such large aperture is typical for high-power devices [36,37]. The resonant cavity is surrounded by 32 pairs of silicon doped n-type DBRs and 24 pairs of carbon doped p-type DBRs made of ${\mathrm{Al}}_{0.2}$${\mathrm{Ga}}_{0.8}$As/${\mathrm{Al}}_{0.9}$${\mathrm{Ga}}_{0.1}$As. In contrast to our previous work [27], we removed the oxidized island from the resonant cavity and placed it inside the subsequent layers of the top DBRs. When analyzing the impact of the island on optical and thermo-electric properties, we changed not only the diameter of the island but also its position. Setting the island further from the resonant cavity makes it possible to increase the diameter to a value greater than that of the electrical aperture (the results of this analysis are presented below). Both ring contacts are made of gold.

Technologies currently under development allow for the oxidation of areas inside laser structures during their processing. It is therefore possible to oxidize selectively buried layers of AlGaAs [28,29,38]. The process starts with the etching of micron holes in the epitaxial structure above the oxidized layer, by the use of standard lithography techniques. Oxidation follows through the holes and spreads inside the AlGaAs layer. The speed of oxidation depends both on the amount of Al in the material and on the thickness of the layer [39]. After the process of oxidation, the next phase of epitaxial growth can be performed and a stack of layers can be grown above the oxidized material. This technology enables the processing of an ARROW VCSEL with an oxide island, such as that described in this paper.

## 4. Impact of an Oxide Island on Current Flow

**Figure 2:**
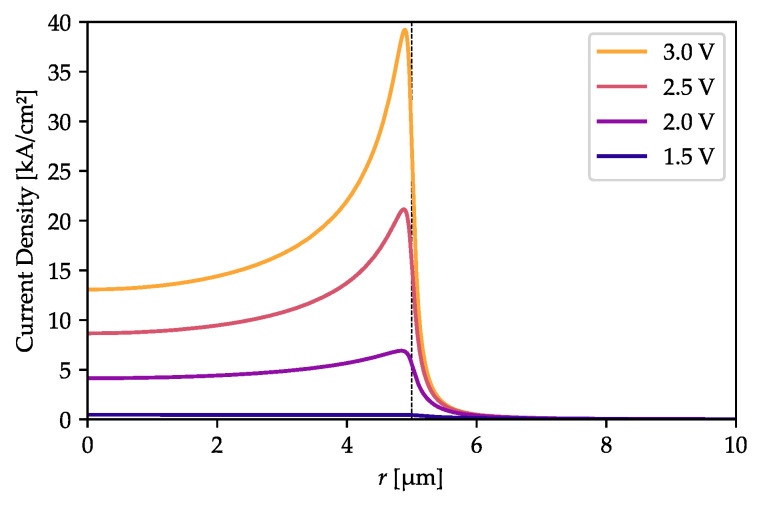
Current density distribution in the active region for several contact-to-contact voltages in a structure without an oxide island. There is some current-crowding at the outer oxidation edges, but only at high voltages. The dashed vertical line indicates the outer oxide aperture radius.

**Figure 3:**
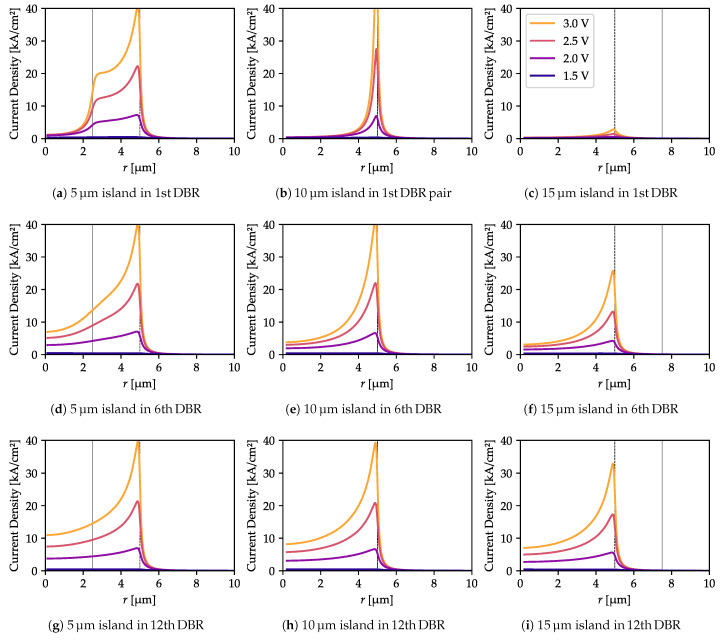
Current density distribution in the active region for several contact-to-contact voltages and with oxide islands of various sizes in different positions. Each figure caption indicates the island diameter and the number of the top DBR pairs in which it is located, counted from the cavity. Current crowding decreases as the island is moved away from the cavity, but becomes larger as the size of the island increases. Large oxide islands strongly increase structure resistivity. Vertical lines indicate the oxide aperture radii (dashed—outer oxidation radius, solid—the island radius).

As can be seen in Figure 1, if the oxide island is located in the lower part of the bottom DBR there is not much space for current flow between the oxidation layers. This should result in very strong current-crowding near the outer oxidation edges. Such an effect can be expected to increase with larger oxide island diameters and to decrease as the distance between the island and the cavity increases (i.e., when the island is located in more distant DBR layers), as this impacts the area at which the current may spread. To confirm this expectation, we computed the current density distributions in the active region for several voltage values. Figure 2 shows these distributions for a structure with no oxide island. Below 2 V, there is only slight current-crowding, increasing to a moderate level at higher voltages. The situation is different with an oxide island (Figure 3). If the island is located close to the cavity (in the 1st DBR pair, Figure 3a–c), it effectively blocks the current flow beneath it. In the case of a moderate distance (in the 6th pair, Figure 3d–f), its impact is still visible. If it is placed in the 12th pair (Figure 3g–i) or above, it has a minor impact on current-crowding (although there is still some).

**Figure 4:**
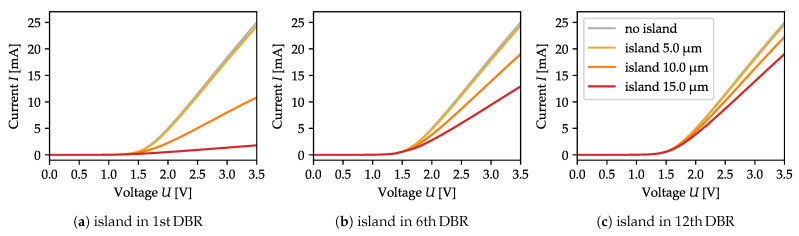
I-V curves for islands located in the 1st, 6th, and 12th DBR pairs for three different oxide island diameters. The impact of the island on structure resistivity is significant if it is close to the cavity and decreases with increasing distance.

The increase in current-crowding results in higher total structure resistivity, which is stronger for an island located closer to the cavity. The I-V curves (Figure 4) reveal that with with the same voltage but larger islands the total current drops. This effect is very strong when the oxide island is located right next to the cavity, and becomes marginal if the island is placed far away. However, when the oxide island has a diameter of 5 µm or less, it has no visible impact on structure resistivity, regardless of its distance from the cavity. On the other hand, it does influence the current density distribution (Figure 3), increasing in value directly outside the island aperture and decreasing in the center (compare Figure 3a,d with the reference current distribution computed in Figure 2, in the absence of an oxide island).

**Figure 5:**
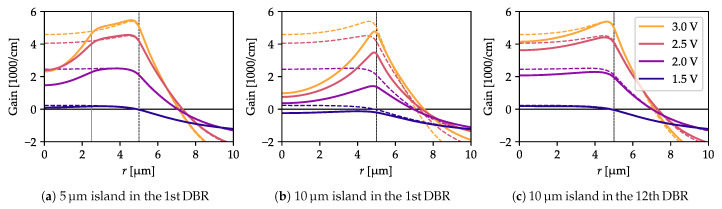
Gain distribution (with spatial hole burning effect neglected) in the active region for several contact-to-contact voltages in a structure with 5 µm (**a**) and 10 µm (**b**,**c**) oxide islands located in the 1st (**a**,**b**) and 12th DBR pair (**c**) compared to the structure with no oxide island at all (thin dashed lines in all plots). The presence of the island influences the gain distribution only if it is located very close to the cavity.

Although—even in the structure with no oxide island—current-crowding is evident for voltages above 2.5 V, the gain distribution is much more uniform due to the carrier diffusion. The gain profile shows a significant dip in the structure center only if the oxide island is located close to the cavity (Figure 5a,b). However, if the the oxide island is located far enough away from the cavity (in the 12th DBR pair in this case), it only slightly influences the gain distribution (Figure 5c). Hence, in this case, the antiresonant effect and its impact on the optical mode thresholds (analyzed in detail in the next section) is not affected by the distorted gain distribution.

It is important to mention that the profiles shown in Figure 5 do not consider the spatial hole burning effect. However, we have intentionally neglected it, as it gives the minimum modes discrepancy and helps to find the optimal structure [40,41,42]. The impact of the spatial hole burning is discussed in Section 5.

## 5. Lasing Conditions for Optical Modes

In order to understand the real impact of the oxide island on the modal behavior of the analyzed VCSEL, we determine threshold currents for lateral modes of different orders for various island diameters and positions. As stated in the introduction, we performed a similar analysis in a previous study for a cold cavity [27], and demonstrated strong oscillations in the optical losses, mainly in the ${\mathrm{HE}}_{11}$ and ${\mathrm{HE}}_{12}$ modes. Although the structure was slightly different than that considered in here—the oxide island was located inside the cavity (instead of in the top DBR) and the top DBR was dielectric—the physical mechanism causing these oscillations (the changing of the mode profiles due the differently sized islands) remains the same. We can therefore expect similar behavior in the current structure. However, this time we take a step further and consider the overlap between the gain and the optical mode profile which is distorted by the island, as well as the impact of the thermal heating caused by the current flow.

**Figure 6:**
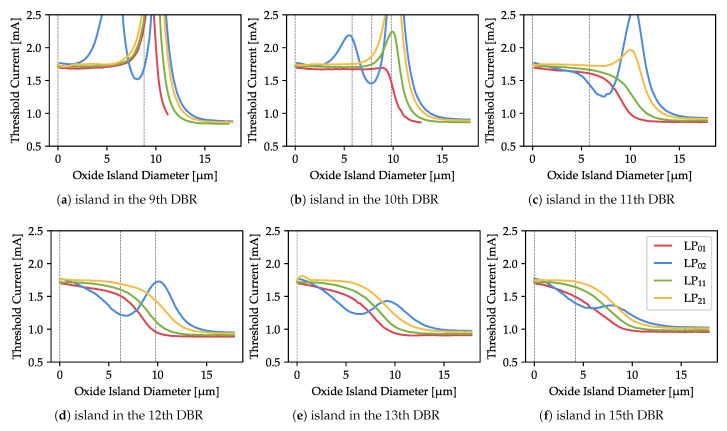
Dependence of threshold current on the size of the oxide island for several locations in the DBR layers. The position of the island has a strong impact the qualitative behavior of the modes. Below the 12th pair, strong oscillations are visible. A rapid increase in threshold current can be observed for critical island diameters (10 µm and around 5 µm for the ${\mathrm{LP}}_{02}$ mode). Dashed gray lines indicate selected parameters for which the optical profiles are analyzed.

A good estimate of these effects is the threshold current, which is computed for each mode separately. We compute the threshold current for the dominant modes (namely ${\mathrm{LP}}_{01}$, ${\mathrm{LP}}_{02}$, ${\mathrm{LP}}_{11}$, and ${\mathrm{LP}}_{21}$) in the case of an island located around the 12th DBR pair. As shown in Figure 4 and Figure 5, this distance is sufficient to avoid a significant increase in structure resistivity and to keep the gain distribution in the active region acceptably uniform. The results are shown in Figure 6. As can clearly be seen, the modal behavior strongly depends on the distance of the oxide island from the cavity. When it is positioned below the 12th DBR pair, there are strong oscillations in the threshold current which increase with the island diameter. For more distant island locations, these oscillations are strongly suppressed and almost disappear.

**Figure 7:**
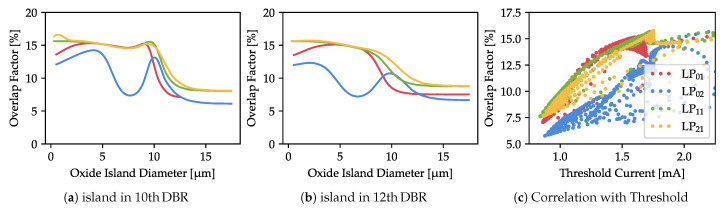
Overlap factor at threshold for the oxide island located in the (**a**) 10th and (**b**) 12th DBR pairs and (**c**) the correlation between the overlap factor and the threshold current for all island distances presented in Figure 6. The oscillations of the threshold current are not correlated with the overlap factor.

For islands positions where oscillations occur, the strongest increase of the threshold current can be observed with diameters of around 10 µm, which is exactly the diameter of the outer oxidation located in the cavity. There are two possible reasons for this: an increase in the optical modal loss, or a decrease in the overlap between the light and the gain. Figure 7a,b show the overlap factor for an island located in the 10th and 12th DBR pairs. This factor is a unitless value that should be 1 if the profile of the gain exactly matches the profile of the optical mode and 0 if there is no overlap at all. It is calculated as

$$\eta =\frac{\int \;P\;g\;r\;dr}{\sqrt{\int \;\;P^{2}\;r\;dr\;\int \;\;g^{2}\;r\;dr}}$$

where *P* is the light intensity distribution, *g* is the material gain, and the integration is performed over the active region. As can be seen from Figure 7c, there is no expected negative correlation between the overlap factor and the threshold. At the 10 µm island (where the threshold is highest), the overlap not only does not decrease but increases visibly to double the size of the overlap for the 15 µm island (for which the threshold is the lowest). This happens for all the analyzed modes. Hence, we conclude that for the analyzed island distances (9th–15th DBR pairs) the overlap between the optical mode and the gain has a negligible impact on the threshold current.

**Figure 8:**
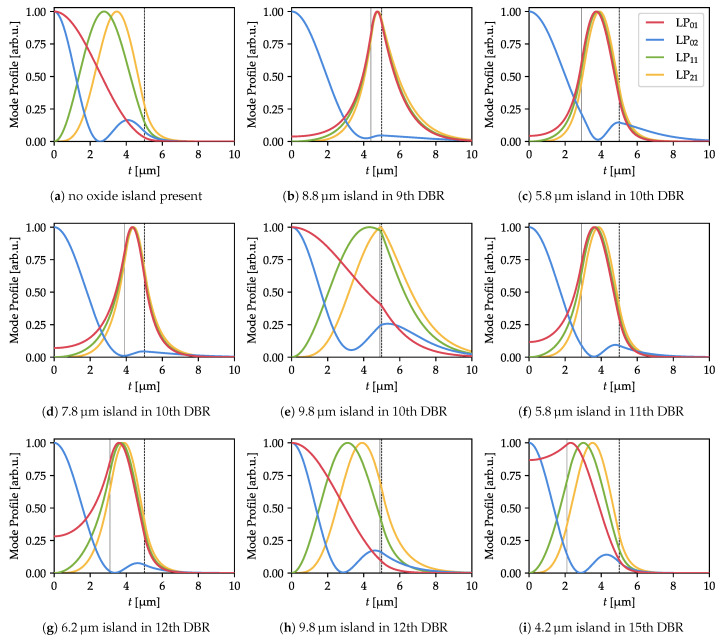
Light intensity profiles for selected island positions and sizes. Dashed vertical black lines indicate the outer aperture radius, while the solid gray line shows the island aperture radius. In points where ${\mathrm{LP}}_{02}$ has a low threshold current, it is mostly located near the device axis. When it has a strong second-order peak its threshold rises.

The oscillations in the threshold current are similar in character to the oscillations of the cold-cavity modal loss we investigated in [27]. There, we explained such oscillations in terms of the match/mismatch of the optical mode profile with the island aperture. In the present work, the situation is different, as the oxide island is located not in the cavity but in the top DBR. However, the origin of the threshold variations is the same: match or mismatch of the modal profile with the antiresonant oxide aperture. As evidence, we present the profiles of the optical modes in selected characteristic points (defined by the size of the oxide island and its vertical position, indicated with vertical gray dashed lines in Figure 6), where the threshold currents presented show peculiar behavior (Figure 8).

Regardless of the position of the island, it forces all ${\mathrm{LP}}_{x1}$ modes to shift their maximum to the high-index region, which for closer islands increases their optical losses and, as a consequence, the threshold current. However, for an island size close to that of the outer aperture and larger, the modes shift back to the center and their profiles resemble those of the modes when there is no oxide island (Figure 8a). As a result, modal losses are reduced. This also explains why the threshold stabilizes for islands significantly larger than the outer aperture.

The ${\mathrm{LP}}_{02}$ mode behaves differently from the other modes. For example, for an island located in the 10th DBR pair, the ${\mathrm{LP}}_{02}$ mode is pushed away to the outer radii (the primary maximum in the center widens and the secondary maximum shifts outside the outer aperture (Figure 8c). This results in a rise in the threshold current. For wider islands, the secondary maximum disappears (Figure 8d) and the threshold drops: it becomes the lowest of all the modes. If the island size reaches the outer aperture, all the modes but ${\mathrm{LP}}_{01}$ have a strong (either primary or secondary) maximum at the aperture edge (Figure 8e) and thus a very high threshold (Figure 6b).

The role of the distance of the island from the cavity can be seen by comparing Figure 8c with Figure 8g,i, which show island sizes of approximately 6 µm. With a closer island, the ${\mathrm{LP}}_{01}$ mode drops almost entirely to 0 at the axis and its profile resembles the profiles of the higher order modes (e.g., ${\mathrm{LP}}_{11}$). When the island is located just two more DBR pairs further away, there is non-negligible light intensity in the device axis. For even further island, the fundamental mode drop in its region is minor (Figure 8i). Similarly, comparison of Figure 8e,h (made for an island of approximately 10 µm) reveals that in the latter case the mode profiles are almost completely undistorted when compared with the island-less structure (Figure 8a). The closer island still strongly distorts the ${\mathrm{LP}}_{21}$ mode, causing its threshold to rise.

**Figure 9:**
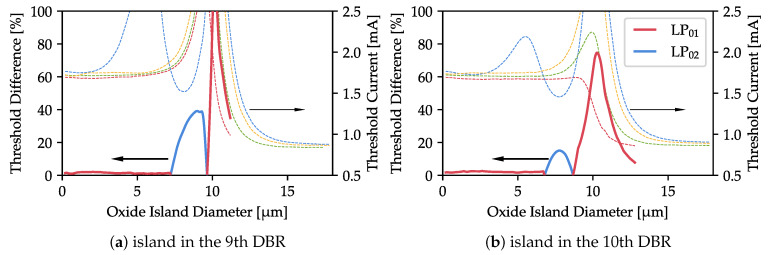
Threshold current differences between the mode with the lowest threshold and that with the next lowest threshold current. The line colors indicate the lowest-threshold mode. For reference, we reproduce threshold currents from Figure 6.

From a practical point of view, the impact of the oxide island on the lasing threshold of different-order modes can be used to ensure single-mode operation of the laser. To achieve this goal, we must ensure that one of the modes is strongly preferred over the others. As the results presented in Figure 6 suggest, this is possible for islands located closer to the cavity, i.e., in the 9th or 10th DBR pair. Figure 9 shows the threshold discrimination in these cases, defined as

$$D=\frac{I_{\mathrm{th}}\left[2\right]-I_{\mathrm{th}}\left[1\right]}{I_{\mathrm{th}}\left[1\right]},$$

where $I_{\mathrm{th}}\left[1\right]$ is the threshold current for the mode with the lowest threshold and $I_{\mathrm{th}}\left[2\right]$ is the threshold current for the second lowest-threshold mode. The colors of the lines in Figure 9 indicate the mode which should start lasing first. It is known [40,41,42] that a large difference in the threshold, caused by a strong discrepancy in the optical losses, favors the lowest-threshold mode in the modal competition and provides single-mode emission in a wide driving current range.

Analysis of Figure 9 provides useful insights regarding the size and location of the oxide island that gives the highest discrimination. As can clearly be seen, an island of approximately 10 µm–11 µm in the 9th DBR pair should be optimal. However, in this case, all the modes are relatively lossy, the fundamental mode has a high threshold, and both the threshold and the discrimination vary very quickly with island size. Taking into account the limited precision of both the numerical analysis and the fabrication process, these parameters are not stable enough to be of practical use.

For an island in the 9th DBR pair, a much safer choice is a diameter of 8.8 µm, for which modal discrimination is 40%. In this case, the lowest threshold mode is ${\mathrm{LP}}_{02}$ and its profile is the most Gaussian-like of all the modes, with the highest maximum located in the device axis (Figure 8b). Much higher discrimination of around 80% can be achieved for a 10 µm island in the 10th DBR pair. The physical origin of the discrimination peak is the same as that of an island in the 9th DBR pair, which we consider impractical. However, in this case the ${\mathrm{LP}}_{01}$ threshold changes much more smoothly and, more importantly, shows no strong rise, as there was in the previously analyzed case. The fundamental mode profile is much wider (Figure 8e) than in the case without an oxide island. This delivers the additional advantage of lower diffraction.

For the optimal design, where *D* is the largest (which corresponds to the 10.2 µm island in the 10th DBR pair) we have investigated the output power for the increasing input current. The device is not single-mode for its whole range of operation, however, it remains single-mode up to the 3.75 mA, which gives emitted power of 2.9 mW. Although this is not a record value for a 850 nm VCSEL (e.g., when compared to [11]), it proves that the oxide island has positive impact on the single-mode mode and we expect it can be improved by tuning design other parameters than the oxide island, which is out of scope of this article.

**Figure 10:**
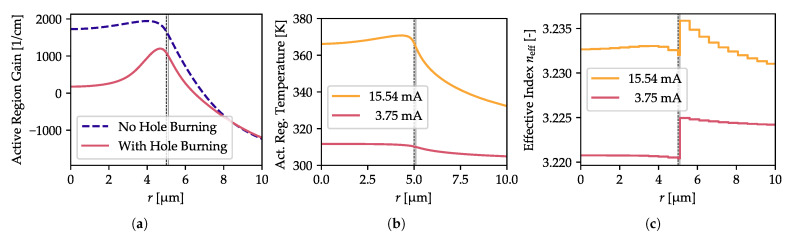
(**a**) Gain profile at 3.75 mA with and without spatial hole burning. (**b**) Temperature profile in the active region for input currents of 3.75 mA and 15.54 mA. (**c**) Effective index distribution for the same input currents. For the lower current, the heating is low and the oxide island has the strongest impact on the effective-index distribution, while for the high current, thermal lensing has strength comparable to that of the oxide island. Visible staircase is a result of the numerical approximations used in our calculations. In all plots, black dashed and solid gray vertical lines indicate the outer aperture and the island radius, respectively.

The input current of 3.75 mA, at which the ${\mathrm{LP}}_{11}$ mode appears is higher than its estimated threshold shown in Figure 6b (2.17 mA). This is caused by the spatial hole burning effect induced by the ${\mathrm{LP}}_{01}$ mode, which was neglected in the earlier calculations. However, it is shown in Figure 10a, which presents comparison of the gain profile at 3.75 mA determined neglecting spatial hole burning and with taking it into consideration.

To maintain a single-mode regime, the impact of the oxide island on the optical properties of the laser must be larger than the thermal lensing effect. Up to the input current of 3.75 mA this is indeed the case, as the heating is not significant: around 12 K over ambient temperature of 300 K, as shown in Figure 10b. In consequence the radial effective-index distribution of the VCSEL shows a strong change only at the island radius (Figure 10c). For comparison, at 15.54 mA, where the laser heats by 70 K, the thermal lensing is strong and the effect of the oxide aperture is reduced (see yellow lines in Figure 10b,c). Hence, the laser switches to the multi-mode regime and (as our calculations show) the total emitted power reaches 10 mW.

## 6. Conclusions

The use of an antiresonant, low-refractive-index oxide island, manufactured by the planar oxidation method and located in the top DBR, can affect both the current flow and the lasing conditions of a VCSEL. Placing the island close to the cavity significantly increases current-crowding (for small island diameters) or the differential resistance (for large diameters). However, if the distance between the cavity and the island is more than 6 DBR pairs, both these effects are reduced and, for an island in the 12th DBR pair, they have a negligible impact on the current flow.

In the analyzed laser, the critical point at which the qualitative behavior of the modal loss and threshold gain change seems be at a distance of 12 DBR pairs. With closer islands, there are strong oscillations in the threshold current which increase with the oxide island diameter and diminish with increasing distances between the island and the cavity. The origin of these oscillations is related to the mode profile and the amount of light outside the outer aperture.

The strong impact of the oxide island on the lasing conditions of different-order modes can be tailored to increase modal discrimination and support single-mode operation of the laser. 

## References

[1] Jung C., Jäger R., Grabherr M., Schnitzer P., Michalzik R., Weigl B., Müller S., Ebeling K. (1997). 4.8 mW Singlemode Oxide Confined Top-Surface Emitting Vertical-Cavity Laser Diodes. Electron. Lett..

[2] Grabherr M., Jager R., Michalzik R., Weigl B., Reiner G., Ebeling K. (1997). Efficient Single-Mode Oxide-Confined GaAs VCSEL’s Emitting in the 850-Nm Wavelength Regime. IEEE Photon. Technol. Lett..

[3] Ueki N., Sakamoto A., Nakamura T., Nakayama H., Sakurai J., Otoma H., Miyamoto Y., Yoshikawa M., Fuse M. (1999). Single-Transverse-Mode 3.4-mW Emission of Oxide-Confined 780-Nm VCSELs. IEEE Photon. Technol. Lett..

[4] Shchukin V., Ledentsov N.N., Kropp J., Steinle G., Ledentsov N., Burger S., Schmidt F. (2014). Single-Mode Vertical Cavity Surface Emitting Laser via Oxide-Aperture-Engineering of Leakage of High-Order Transverse Modes. IEEE J. Quantum Electron..

[5] Stepniak G., Lewandowski A., Kropp J., Ledentsov N., Shchukin V., Ledentsov N., Schaefer G., Agustin M., Turkiewicz J. (2016). 54 Gbit/s OOK Transmission Using Single-Mode VCSEL up to 2.2 Km MMF. Electron. Lett..

[6] Puerta R., Agustin M., Chorchos Ł., Toński J., Kropp J.R., Ledentsov N., Shchukin V.A., Ledentsov N.N., Henker R., Monroy I.T. 107.5 Gb/s 850 Nm Multi- and Single-Mode VCSEL Transmission over 10 and 100 m of Multi-Mode Fiber. Proceedings of the 2016 Optical Fiber Communications Conference and Exhibition (OFC).

[7] Kao H.Y., Tsai C.T., Chi Y.C., Peng C.Y., Leong S.F., Wang H.Y., Cheng C.H., Wu W.L., Kuo H.C., Cheng W.H. (2019). Long-Term Thermal Stability of Single-Mode VCSEL Under 96-Gbit/s OFDM Transmission. IEEE J. Sel. Top. Quantum Electron..

[8] Guan B., Li P., Arafin S., Alaskar Y., Wang K.L. (2018). Investigation of Single-Mode Vertical-Cavity Surface-Emitting Lasers with Graphene-Bubble Dielectric DBR. Photonics Nanostructures Fundam. Appl..

[9] Ledentsov N., Turkiewicz J.P., Chorchos Ł., Ledentsov N.N., Agustin M. Leaky Cavity 850 Nm Single-Mode VCSELs for High-Speed Data Transmission over Multi-Mode Fiber. Proceedings of the 2018 Photonics in Switching and Computing (PSC).

[10] Haglund Å., Gustavsson J.S., Vukušić J., Modh P., Larsson A. (2004). Single Fundamental-Mode Output Power Exceeding 6 mW From VCSELs With a Shallow Surface Relief. IEEE Photonics Technol. Lett..

[11] Shi J.W., Wei Z.R., Chi K.L., Jiang J.W., Wun J.M., Lu I.C., Chen J., Yang Y.J. (2013). Single-Mode, High-Speed, and High-Power Vertical-Cavity Surface-Emitting Lasers at 850 Nm for Short to Medium Reach (2 Km) Optical Interconnects. J. Light. Technol..

[12] Khan Z., Shih J.C., Cheng C.L., Shi J.W. High-Power and Highly Single-Mode Zn-Diffusion VCSELs at 940 Nm Wavelength. Proceedings of the 2019 IEEE Photonics Conference (IPC).

[13] Bao L., Kim N.H., Mawst L.J., Elkin N.N., Troshchieva V.N., Vysotsky D.V., Napartovich A.P. (2007). Single-Mode Emission From Vertical-Cavity Surface-Emitting Lasers With Low-Index Defects. IEEE Photon. Technol. Lett..

[14] Dems M., Beling P., Gębski M., Piskorski Ł., Walczak J., Kuc M., Frasunkiewicz L., Michał  W., Sarzała R., Czyszanowski T. (2015). VCSEL Modeling with Self-Consistent Models: From Simple Approximations to Comprehensive Numerical Analysis. Proc. SPIE.

[15] Haglund E., Jahed M., Gustavsson J.S., Larsson A., Goyvaerts J., Baets R., Roelkens G., Rensing M., O’Brien P. (2019). High-Power Single Transverse and Polarization Mode VCSEL for Silicon Photonics Integration. Opt. Express.

[16] Kokubun Y., Baba T., Sakaki T., Iga K. (1986). Low-Loss Antiresonant Reflecting Optical Waveguide on Si Substrate in Visible-Wavelength Region. Electron. Lett..

[17] Koch T., Koren U., Boyd G., Corvini P., Duguay M. (1987). Antiresonant Reflecting Optical Waveguides for III-V Integrated Optics. Electron. Lett..

[18] Yin D., Schmidt H., Barber J.P., Hawkins A.R. (2004). Integrated ARROW Waveguides with Hollow Cores. Opt. Express.

[19] Ledentsov N.N., Shchukin V.A., Kalosha V.P., Ledentsov N.N., Kropp J.R., Agustin M., Chorchos Ł., Stępniak G., Turkiewicz J.P., Shi J.W. (2018). Anti-Waveguiding Vertical-Cavity Surface-Emitting Laser at 850 Nm: From Concept to Advances in High-Speed Data Transmission. Opt. Express.

[20] Wu Y., Li G., Nabiev R., Choquette K., Caneau C., Chang-Hasnain C. (1995). Single-Mode, Passive Antiguide Vertical Cavity Surface Emitting Laser. IEEE J. Sel. Top. Quantum Electron..

[21] Goltser I.V., Mawst L.J., Botez D. (1995). Single-Cladding Antiresonant Reflecting Optical Waveguide-Type Diode Laser. Opt. Lett. OL.

[22] Zhou D., Mawst L.J. (2002). High-Power Single-Mode Antiresonant Reflecting Optical Waveguide-Type Vertical-Cavity Surface-Emitting Lasers. IEEE J. Quantum Electron..

[23] Tee C.W., Yu S.F. (2003). Design and Analysis of Cylindrical Antiresonant Reflecting Optical Waveguide. J. Light. Technol..

[24] Tee C., Tan C., Yu S. (2003). Design of Antiresonant-Reflecting Optical Waveguide-Type Vertical-Cavity Surface-Emitting Lasers Using Transfer Matrix Method. IEEE Photonics Technol. Lett..

[25] Tee C., Yu S., Chen N. (2004). Transverse-Leaky-Mode Characteristics of ARROW VCSELs. J. Light. Technol..

[26] Tee C.W., Yu S.F., Penty R.V., White I.H. (2005). Transient Response of ARROW VCSELs. IEEE J. Quantum Electron..

[27] Więckowska M., Czyszanowski T., Almuneau G., Dems M. (2018). Shaping Vertical-Cavity Surface-Emitting Laser Mode Profiles with an Antiresonant Oxide Island for Improved Single-Mode Emission. J. Opt. Soc. Am. B.

[28] Amat C., Almuneau G., Gallo P., Jalabert L., Moumdji S., Dubreuil P., Camps T., Doucet J.B., Havard E., Bardinal V. (2007). Free Engineering of Buried Oxide Patterns in GaAs/AlAs Epitaxial Structures. Electron. Lett..

[29] Chouchane F., Doucet J.B., Arnoult A., Lacoste G., Fontaine C., Almuneau G. (2012). A New Approach of Planar Oxidation of Buried Al xGa 1-xAs/GaAs Epitaxial Structures for Optical and Electrical Confinement Applications. Phys. Status Solidi Curr. Top. Solid State Phys..

[30] Sarzała R., Czyszanowski T., Wasiak M., Dems M., Piskorski L., Nakwaski W., Panajotov K. (2012). Numerical Self-Consistent Analysis of VCSELs. Adv. Opt. Technol..

[31] Zeghuzi A., Wenzel H., Wünsche H.J., Radziunas M., Bandelow U., Knigge A., Osiński M., Arakawa Y., Witzigmann B. (2018). Modeling of Current Spreading in High-Power Broad-Area Lasers and Its Impact on the Lateral Far Field Divergence. Physics and Simulation of Optoelectronic Devices XXVI.

[32] Radziunas M., Fuhrmann J., Zeghuzi A., Wünsche H.J., Koprucki T., Brée C., Wenzel H., Bandelow U. (2019). Efficient Coupling of Dynamic Electro-Optical and Heat-Transport Models for High-Power Broad-Area Semiconductor Lasers. Opt. Quant. Electron..

[33] Piskorski Ł., Sarzała R.P., Nakwaski W. (2007). Self-Consistent Model of 650 Nm GaInP/AlGaInP Quantum-Well Vertical-Cavity Surface-Emitting Diode Lasers. Semicond. Sci. Technol..

[34] Dems M., Kotynski R., Panajotov K. (2005). Plane Wave Admittance Method—A Novel Approach for Determining the Electromagnetic Modes in Photonic Structures. Opt. Express.

[35] Wenzel H., Wünsche H.J. (1997). The Effective Frequency Method in the Analysis of Vertical-Cavity Surface-Emitting Lasers. IEEE J. Quantum Electron..

[36] Li T., Hao E.J. High Performance 850nm VCSELs with Surface Relief. Proceedings of the 2010 Academic Symposium on Optoelectronics and Microelectronics Technology and 10th Chinese-Russian Symposium on Laser Physics and Laser TechnologyOptoelectronics Technology (ASOT).

[37] Moser P. (2016). Energy-Efficient VCSELs for Optical Interconnects.

[38] Calvez S., Calmon P.F., Arnoult A., Gauthier-Lafaye O., Fontaine C., Almuneau G. (2017). Low-Loss Buried AlGaAs/AlOx Waveguides Using a Quasi-Planar Process. Opt. Express.

[39] Suarez I., Condé M., Bouscayrol L., Fontaine C., Almuneau G. (2008). Structure-Induced Effects on the Selective Wet Thermal Oxidation of Digital Al*_x_* Ga_1–*x*_ As Alloy. J. Mater. Res..

[40] Czyszanowski T., Dems M., Sarzała R.P., Nakwaski W., Panajotov K. (2011). Precise Lateral Mode Control in Photonic Crystal Vertical-Cavity Surface-Emitting Lasers. IEEE J. Quantum Electron..

[41] Czyszanowski T., Sarzała R.P., Dems M., Walczak J., Wasiak M., Nakwaski W., Iakovlev V., Volet N., Kapon E. (2013). Spatial-Mode Discrimination in Guided and Antiguided Arrays of Long-Wavelength VCSELs. IEEE J. Sel. Top. Quantum Electron..

[42] Czyszanowski T., Volet N., Walczak J., Dems M., Sarzala R.P., Iakovlev V., Sirbu A., Mereuta A., Caliman A., Kapon E. (2014). Numerical Analysis of Mode Discrimination by Intracavity Patterning in Long-Wavelength Wafer-Fused Vertical-Cavity Surface-Emitting Lasers. IEEE J. Quantum Electron..

